# Intracranial Virotherapy for a Canine Hemangioma

**DOI:** 10.3390/ijms231911677

**Published:** 2022-10-02

**Authors:** Pablo Delgado-Bonet, Beatriz Davinia Tomeo-Martín, Blanca Delgado-Bonet, David Sardón-Ruiz, Angel Torrado-Carvajal, Isidro Mateo, Ana Judith Perisé-Barrios

**Affiliations:** 1 Biomedical Research Unit (UIB-UAX), Universidad Alfonso X el Sabio, 28691 Madrid, Spain; 2 Medical Image Analysis and Biometry Laboratory, Universidad Rey Juan Carlos, 28933 Madrid, Spain; 3 Department of Veterinary Pathology, Universidad Alfonso X el Sabio, 28691 Madrid, Spain; 4 Neurology Service, Veterinary Hospital VETSIA, 28914 Madrid, Spain; 5 Neurology Service, Veterinary Clinical Hospital, Universidad Alfonso X el Sabio, 28691 Madrid, Spain

**Keywords:** ICOCAV15, oncolytic adenovirus, virotherapy, immunotherapy, hemangioma, brain tumor, volumetric criteria

## Abstract

Intracranial hemangiomas are rare neoplastic lesions in dogs that usually appear with life-threatening symptoms. The treatment of choice is tumor resection; however, complete resection is rarely achieved. The patient’s prognosis therefore usually worsens due to tumor progression, and adjuvant treatments are required to control the disease. Oncolytic viruses are an innovative approach that lyses the tumor cells and induces immune responses. Here, we report the intratumoral inoculation of ICOCAV15 (an oncolytic adenovirus) in a canine intracranial hemangioma, as adjuvant treatment for incomplete tumor resection. The canine patient showed no side effects, and the tumor volume decreased over the 12 months after the treatment, as measured by magnetic resonance imaging using volumetric criteria. When progressive disease was detected at month 18, a new dose of ICOCAV15 was administered. The patient died 31.9 months after the first inoculation of the oncolytic adenovirus. Furthermore, tumor-infiltrated immune cells increased in number after the viral administrations, suggesting tumor microenvironment activation. The increased number of infiltrated immune cells, the long survival time and the absence of side effects suggest that ICOCAV15 could be a safe and effective treatment and should be further explored as a novel therapy for canine hemangiomas.

## 1. Introduction

Dogs have the highest incidence of brain tumors among pets, with an unclear prognosis and limited treatment options [1,2]. Although meningiomas and gliomas are the most frequent primary intracranial tumors reported in dogs, vascular tumors can also appear with similar symptoms [3]. Canine hemangiomas are benign neoplastic proliferative lesions of endothelial origin, most frequently found subcutaneously, and they can usually be cured after complete surgical excision [4,5]. Primary detection of this neoplasm in the nervous system is rare, and only a few reports on canine neuro-oncology have been published [6,7,8,9,10]. Several authors have suggested that the low incidence of this neoplasm in veterinary medicine could be associated with a lack of well-established criteria to discriminate between neoplastic lesions and hamartomas [11]. 

The reported incidence of canine hemangiomas in the central nervous system is low; nevertheless, the treatment of choice is based on the human medical approach, in which complete surgical resection of the tumor is proposed [12,13,14]. In veterinary medicine, complete surgical excision has been reported in two dogs: an intracranial and an intramedullary hemangioma, achieving complete responses for 13 and 22 months, respectively [6,15]. When complete tumor excision is not a feasible option, the likelihood of hemorrhages and relapses increases, and adjuvant therapies such as radiotherapy are indicated [16,17,18,19]. The limited access to other treatments for intracranial tumors in veterinary medicine necessitates research to discover alternative adjuvant therapies. Oncolytic virotherapy could be a therapeutic option for canine spontaneous tumors, with several trials showing promising response rates with good tolerability [20,21,22,23,24]. Recently, a veterinary trial using the oncolytic adenovirus ICOCAV15 to treat cutaneous tumors has shown clinical benefit, with no adverse effects due to the virotherapy [25]. Additionally, various clinical studies using adenovirus to treat canine gliomas and human intracranial tumors shown no adverse effects, suggesting that oncolytic adenovirus could safely treat central nervous system tumors [21,26,27,28]. In this study, we describe the use of the oncolytic adenovirus ICOCAV15 as adjuvant therapy for an incomplete surgical resection of a canine intracranial hemangioma.

## 2. Results

### 2.1. Follow-Up and Safety Evaluation

**Figure 1:**
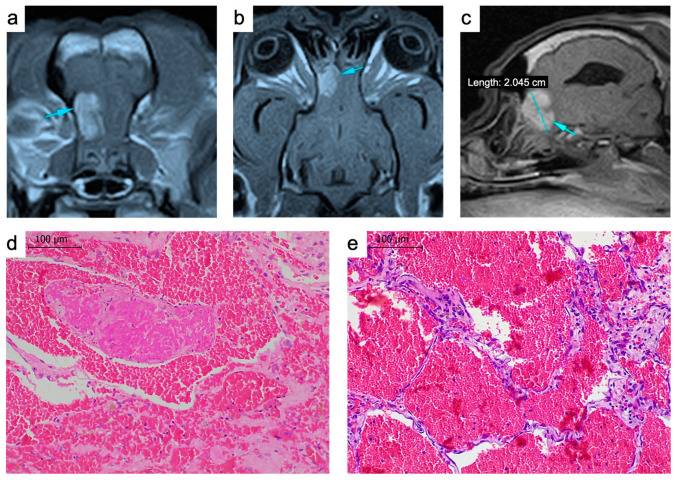
(**a**–**c**) Magnetic resonance images of the tumor. The T1-weighted sequence used for the diagnosis confirming an intra-axial trabeculated mass (arrows) in the left olfactory bulb; transversal (**a**), coronal (**b**) and sagittal (**c**) images. (**d**,**e**) Hematoxylin and eosin-stained tumor biopsy. Multiple thin vessels replete with blood and thromboses (**d**). Scale bar: 100 μm.

A nine-year-old spayed female French bulldog showed an acute appearance of seizures. The clinical and neurological examination, hematological parameters and serum biochemistry revealed no significant findings. Abdominal ultrasound and thoracic radiographs were performed, but no pathological signs were observed. Magnetic resonance imaging (MRI) of the head was performed, which detected a trabeculated intra-axial lesion in the left olfactory bulb. The mass had a hyperintense signal in the T2-weighted and FLAIR sequences and an isointense signal in the T1-weighted sequence. The post-contrast T1-weighted images showed intense and heterogenous uptake in the lesion area; therefore, the first presumptive diagnosis was an intracranial primary tumor (Figure 1a–c). The diagnosis was confirmed by histopathologic analysis of a tumor biopsy obtained by craniotomy. We observed a neoplastic proliferation with well-differentiated, ectatic blood vessels lined by a single layer of flattened endothelial cells with multiple thromboses and multifocal hemorrhages, with no signs of malignant cell proliferation (Figure 1d,e), findings that suggested an intracranial hemangioma.

**Figure 2:**
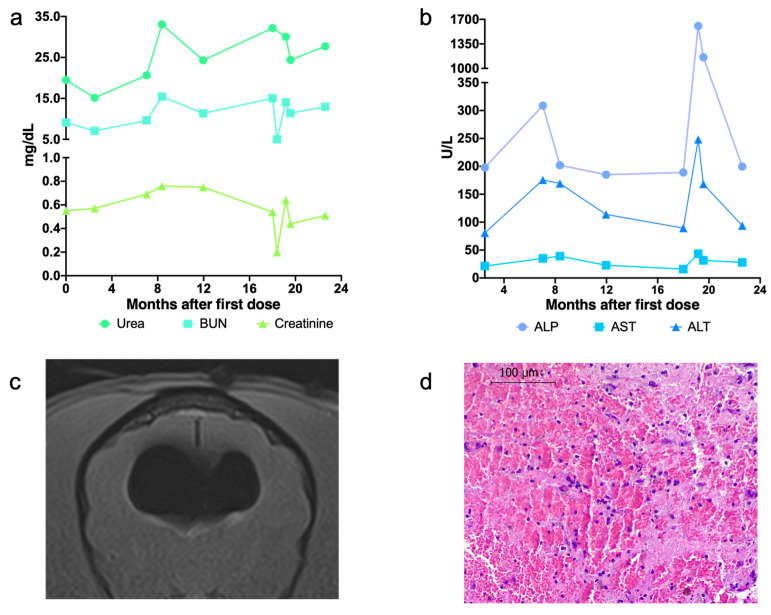
(**a**,**b**) Peripheral blood parameters during follow-up. Quantification of renal biomarkers (**a**) and hepatic enzymes (**b**) in peripheral blood. (**c**) Pneumocephalus image by MRI. Image from 15 days after the second craniotomy. (**d**) Tumor tissue at necropsy. Image of hematoxylin-eosin stained tissue. Scale bar: 100 μm. BUN, blood urea nitrogen; ALP, alkaline phosphatase; AST, aspartate aminotransferase; ALT, alanine transaminase.

**Figure 3:**
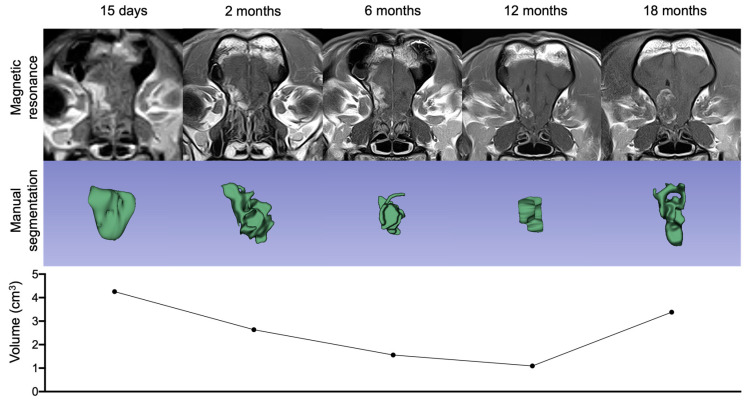
Magnetic resonance follow-up and volumetric response assessment. Representation of the T1-weighted postcontrast transverse sequence, the tumor imaged manual segmentations and volumetric measurements during follow-up.

A transfrontal bilateral craniotomy to resect the tumor and a subsequent inoculation of the oncolytic virus were performed without complications. Seventy-four hours after the ICOCAV15 inoculation, the canine patient was discharged with a maintenance treatment of phenobarbital and a tapering course of corticoids. After the first administration of the oncolytic virus, no significant changes were observed in the physical and neurological examination. During the follow-up, the peripheral blood analysis was normal (Figure 2a,b). Eighteen months later, tumor progression was detected by MRI (Figure 3). Considering the positive clinical response obtained after the first administration according to the neuro-oncologist veterinarian and with the dog owner’s consent, a second surgery with re-inoculation of the oncolytic adenovirus ICOCAV15 was performed. As with the first surgery, no complications were observed after 72 h, and the dog was discharged with a tapering course of corticoids and maintenance phenobarbital. Fifteen days after the second surgery the dog showed signs of generalized pain and depression, and a new MRI showed rounded dilation of the lateral ventricles filled with air (Figure 2c). The peripheral blood analysis at this time showed increased levels of hepatic enzymes, alkaline phosphatase and alanine transaminase, which slowly normalized after withdrawing the corticoids (Figure 2b). After this complication, the dog’s owners decided not to continue with the planned follow-up revisions. Thirty-one months after the diagnosis, the dog died due to epileptic seizures. Histopathology analysis of the necropsy sample revealed focal hemorrhages in the nervous tissue surrounding the intracranial hemangioma, most likely due to an acute hemorrhage of the primary tumor (Figure 2d).

### 2.2. Outcome Assessment by Imaging Technology

To assess the tumor volume and the clinical response to the treatment, a follow-up MRI was performed. Fifteen days after the first surgery, MRI was used as the baseline to measure the ICOCAV15 effect compared with the subsequent follow-up MRI (Figure 3). From day 15 to month 12 after the injection, there was a gradual reduction in tumor size. According to volumetric criteria, stable disease (SD) was observed for 6 months after the treatment, and partial remission was achieved at month 12 with a 65% reduction in tumor volume (Figure 3). After 18 months, an increase in tumor volume was detected (Figure 3), indicating progressive disease. Given that no additional follow-up images were obtained after the second craniotomy, it was not possible to assess the clinical response to the oncolytic virotherapy. The dog’s overall survival time was 994 days after the initial diagnosis, with a disease-free progression of 12 months after inoculation with ICOCAV15.

### 2.3. Immune Response Evaluation

**Figure 4:**
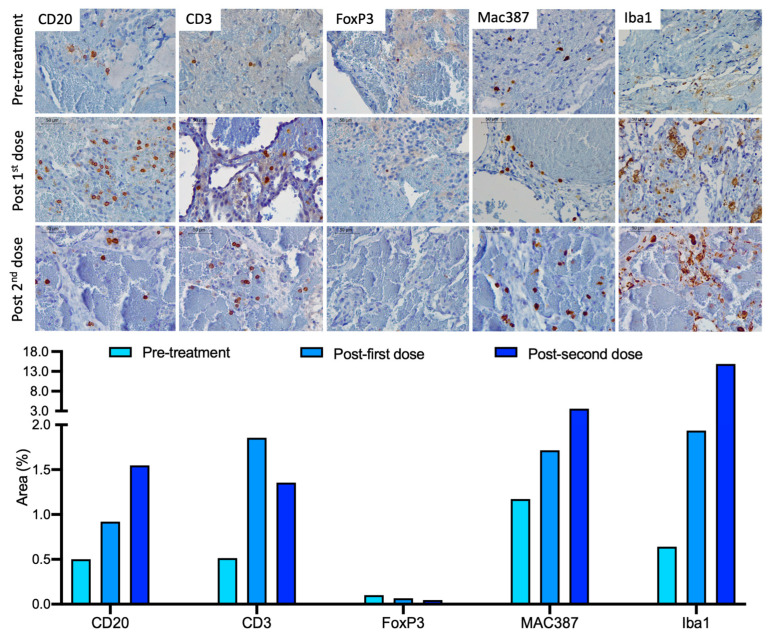
Infiltrated immune cells in the tumor. Tumor tissue pre-treatment (obtained during the first craniotomy), post-first dose (obtained during the second craniotomy) and post-second dose (obtained during necropsy) (top to bottom in each row) were evaluated for the infiltration of B cells (CD20+), T cells (CD3+), regulatory T cells (FoxP3+), M1 macrophages (Mac387+) and activated microglia/macrophages (Iba1+). Scale bar: 50 μm. The graphic representation shows the area (%) of the positive cells (brown) for each marker.

The immune cells infiltrating the tumor were evaluated after both treatments by immunohistochemistry. The evaluated tumor tissues were obtained by biopsy during the first craniotomy (pre-treatment sample), at the second craniotomy (18.6 months after treatment) and during the necropsy (13.3 months after the second inoculation). There was a general increase in the infiltrated immune cells in the tumor after the administration of ICOCAV15 (Figure 4). The number of infiltrated B cells (CD20+) increased after each administration. The number of infiltrated T cells (CD3+) increased after the first dose of ICOCAV15 (1.856%) and in the tumor tissue obtained during necropsy (1.354%) compared with the pre-treatment tissue (0.514%) (Figure 4). A slight decrease in this cell population was observed after the second treatment. The number of regulatory T cells (FoxP3+) decreased after each of the ICOCAV15 doses. Regarding myeloid cells, M1 macrophages (Mac387+) and activated microglia/macrophages (Iba1+) showed a greater area of infiltration, with a tendency to increase in number after each treatment with the oncolytic virus.

### 2.4. ICOCAV and Antiviral Response

The presence of adenoviral DNA from ICOCAV15 could not be detected by quantitative polymerase chain reaction (qPCR) in any of the peripheral blood samples obtained during post-treatment follow-up (from the day after surgery to one month after the second inoculation) or the cerebral spinal fluid (CSF) samples analyzed at day 21 and months 6, 12 and 18 post-treatment. Lastly, viral particles of ICOCAV15 were not detected by qPCR or by immunohistochemistry in the necropsy tissue samples, including the tumor tissue (brain tissue) and distant tissues (liver, spleen and kidneys). Peripheral blood showed no changes in anti-CAV2 antibodies after the administration of the oncolytic adenovirus.

## 3. Discussion

Intracranial hemangiomas are benign vascular tumors rarely reported in dogs. There is insufficient evidence to establish a gold standard treatment but following the human medical literature and limited veterinary case reports, complete surgical resection can achieve long survival [6,14,15]. If the hemangioma is unsuitable for complete excision, acute hemorrhages and tumor progression are possible, leading to a severe deterioration of quality of life [19]. The administration of oncolytic adenovirus has shown beneficial clinical responses in canine and human clinical trials and could improve clinical outcomes as adjuvant therapy for the incomplete excision of intracranial hemangiomas [21,24,25,27,29]. In the present case report, we describe the use and clinical outcome of the adjuvant intratumoral administration of ICOCAV15 in a nine-year-old dog with an intracranial hemangioma.

In terms of the adverse effects of the craniotomy followed by the intracranial administration of ICOCAV15, the dog showed generalized pain after the second surgery due to the presence of a pneumocephalus. This finding was observed in the MRI 15 days after the second intervention, was a consequence of the surgery and is commonly reported after craniectomies in human medicine and in several veterinary reports [30,31,32]. In the present case, we suspected that the air entered from the nasal cavity. The effect was transitory and was controlled by increasing the dosage of corticoids for a short period. Considering that high doses of corticoids can increase liver transaminase levels and that the post-mortem analysis of the liver tissue showed no ICOCAV15 (which could be replicating in the hepatic tissue leading to hepatocellular lysis), the high levels of alkaline phosphatase and aspartate aminotransferase detected in the peripheral blood could be due to the treatment and not to the virus administration [33]. The remaining neurological examinations and parameters analyzed showed no relevant changes after the adjuvant inoculation of ICOCAV15.

The typical criteria implemented in veterinary medicine to measure treatment response is the Response Assessment in Veterinary Neuro-Oncology (RAVNO) based on 2D images. Recently, however, several studies have shown that the volumetric response is more accurate in detecting changes in the tumor burden [34]. Although there was an evident decrease in tumor volume from day 15 after administering the oncolytic virus until month 6, the disease should have been considered a stable disease (SD) according to volumetric criteria. Twelve months after treatment, the volume was reduced by 65%, which was then considered partial response (PR). Eighteen months after the surgical procedure, progressive disease (PD) was detected, and a new surgery and virus inoculation were performed, after which it was not possible to perform a new MRI. It was therefore not possible to assess the progression radiologically after the second dose. Establishing survival times for canine patients with intracranial hemangiomas is difficult because these types of tumors are rare in veterinary medicine and are usually diagnosed during necropsies [10,35]. One case report of complete surgical excision of a canine intracranial hemangioma achieved a survival free-progression time of 13 months after surgical excision. In the present dog treated with the oncolytic virus as adjuvant therapy after incomplete excision of the tumor, the survival time was 31.9 months after two procedures, with a survival free-progression time of 18 and 13.3 months after the first and second dose, respectively [6,8].

After each intracranial administration of ICOCAV15, the infiltration of MAC387+ and Iba1+ cells in the tumor increased, suggesting that ICOCAV15 can induce a change in the tumor microenvironment (TME) [36]. M1 macrophages (MAC387+) and activated microglia (Iba1+) have a proinflammatory behavior and trigger antitumoral responses; therefore, an increased number of these cells could favor a more immunoreactive TME [37]. Furthermore, the increase in the infiltration of CD3+ (reduction in regulatory FoxP3+ subset) and CD20+ lymphocytes after the administration of ICOCAV15 might support the TME’s proinflammatory/antitumor state. The CD3+ lymphocytes were the only evaluated population that presented a higher infiltration after the first administration than in the necropsy. It is possible that the higher infiltration of CD3+ was associated with a better prognosis, given that after the first inoculation the dog was progression-free for 18 months, compared to the 14.7 months after the second administration when the dog presented seizures and died [38]. ICOCAV15 was not detected in the peripheral blood or CSF samples, in contrast to previous reports of oncologic dogs with extracranially treated tumors, suggesting that the blood-brain barrier can reduce the amount of oncolytic adenovirus released to the blood-stream [25]. This finding is relevant given that the release of the oncolytic virus into the blood-stream might elicit antiviral immune responses (through virus-recognizing antibodies), which could reduce the viral load in the tumor [21,25]. 

This is the first time that the outcome of a canine intracranial hemangioma treated with incomplete surgical excision and ICOCAV15 as an adjuvant therapy has been reported. In the present dog, the intracranial inoculation of ICOCAV15 was safe and had a clinical benefit, at least for 18 months. Considering that this is a reported case of intracranial hemangioma and that a beneficial response could be suggested due to the oncolytic virus effect, it should be evaluated in a larger population study.

## 4. Materials and Methods

### 4.1. Diagnosis and Imaging

The presumptive diagnosis was performed by MRI and assessed by a board-approved neurologist. The MRI was performed with two separate devices; the first study was acquired in a Hitachi Airis Mate 0.2T MR scanner (Hitachi Medical AG, Tokyo, Japan) while the following four follow-up studies were performed in an upgraded Toshiba Vantage ELAN 1.5T MR scanner (Toshiba Medical Systems, Tokyo, Japan). The dog was anesthetized and positioned prone to perform the acquisition, trying to maintain the same positioning between studies. The MRI included T2-weighted, T2-FLAIR and T1-weighted (with and without gadolinium enhancement), among others. All sequences were obtained in 2D. The definitive diagnosis was reached by a histopathological analysis of the tumor biopsies obtained during the craniotomies.

### 4.2. Craniotomy, Virotherapy and Medical Treatment

For the craniotomy, the dog was administered (as premedication) intravenous methadone (0.3 mg/kg) and co-induced using lidocaine (1 mg/kg), midazolam (0.2 mg/kg) and propofol (6 mg/kg). The patient was then intubated and anesthetized following total intravenous anesthesia using propofol (0.3–0.7 mg/kg/min) and fentanyl (0.3–0.7 μg/kg/min). A routine transfrontal bilateral craniotomy was performed to achieve partial resection of the mass (biopsy for diagnosis) located in the left olfactory bulb of the frontal lobe. After the incomplete extraction and based on previous work, 10^8^ infectious units of ICOCAV15 were injected into the remaining tissue, as compassionate treatment. The adenoviral particles were diluted in a total volume of 250 µL and then injected in 5 different sites of the tumor. After each procedure, the dog was administered a tapering course of prednisone over 4 weeks. The dosage started at 0.5 mg/kg/12 h for 1 week and lowered to 0.25 mg/kg/12 h, 0.25 mg/kg/24 h and 0.25 mg/kg/48 h the following weeks. Moreover, 2.5 mg/kg/12 h of phenobarbital was initiated and continued during the entire treatment. Prednisone and phenobarbital doses were adjusted during treatment by the veterinary board based on the patient’s needs.

### 4.3. Adverse Effects and Safety

To evaluate any possible adverse effects, blood samples were collected using heparin and EDTA tubes and processed to obtain plasma and cells for flow cytometry analysis. The following parameters were evaluated: hematocrit, hemoglobin, mean corpuscular volume, mean corpuscular hemoglobin concentration, mean corpuscular hemoglobin, red cell blood distribution width, reticulocytes, neutrophils, lymphocytes, monocytes, eosinophils, basophils, platelets, mean platelet volume, platelet distribution width, ions (magnesium, sodium, potassium, chloride, calcium, phosphate), renal parameters (urea, blood urea nitrogen, creatinine), hepatic transaminases (alkaline phosphatase, aspartate aminotransferase, alanine transaminase), total proteins (albumin, globulin and albumin/globulin ratio), coagulation times (time of prothrombin, partial time of activated thromboplastin, fibrinogen) and baseline glucose. In each follow-up visit, the patient was evaluated by the board-certified neurologist of the VETSIA veterinary hospital to ensure the patient’s well-being and detect any possible symptoms due to the treatment or disease progression.

### 4.4. Virotherapy Efficacy 

MRI acquisitions were performed on day 15, at 2 months and every 6 months after each administration of the oncolytic virus to evaluate tumor response, using a 1.5 T magnetic resonance. Tumor segmentations were manually delimited from the T1-weighted post-contrast images using 3D Slicer Software (version 5.0.2). The mesh volume (the sum of all tetrahedrons formed from the origin of the region of interest to its boundary) was calculated using PyRadiomics (version 3.0.1), an open-source Python package [39]. The tumor response was evaluated following the volumetric response criteria method adapted from human medicine and described in veterinary medicine [34,40]. The tumor volume on day 15 was taken as the baseline to observe the potential beneficial effect of ICOCAV15, assuming the decrease observed in the first post-procedure MRI was due to the surgery. Complete response (CR) was considered stable or improved clinical status, patient not being administered steroids, the elimination of all enhancing tumor, and elimination of all T2W/FLAIR lesion burden. Partial response (PR) was considered stable or improved clinical status, stable or decrease steroid dose, ≥65% decrease in enhancing tumor or T2W/FLAIR lesion burden. Stable disease (SD) was considered stable or improved clinical status, stable or decrease steroid dose, <65% decrease or <40% increase in enhancing tumor or T2W/FLAIR lesion burden. Progressive disease (PD) was considered clinical deterioration and new neurological signs, ≥40% increase in enhancing tumor or T2W/FLAIR lesion burden [34].

### 4.5. Histopathology and Immunohistochemistry

A routine necropsy was performed at the time of death to obtain tissues from the tumor, brain, liver, spleen, and kidneys. The canine biopsies and necropsy tissues were fixed in 10% formalin for preservation and embedded in paraffin. Five-mm sections were cut with a microtome and dewaxed then rehydrated using an alcohol battery (xylol 2 × 5 min, ethanol 100% 2 × 5 min, ethanol 96% 1 × 5 min, ethanol 70% 1 × 5 min) for hematoxylin-eosin staining or immunostaining. To perform the immunostaining, a previous antigen retrieval was performed with citrate buffer in a pressure cooker (3 min). Two washes were performed with hydrogen peroxide (6%) for 10 min each, followed by a wash of PBS with 0.1% triton to inhibit the endogenous peroxidase. The immunostaining was performed with the VECTOR, R.T.U. VECTASTAIN Kit, using the normal horse serum of the kit for blocking. After this step, the sample was incubated overnight at 4 °C with the primary antibody [anti-CD3 (UCHT1 3 mg/mL, Dako), anti-CD20 (Polyclonal 0.17 μg/mL, Invitrogen), anti-FoxP3 (eBio7979 10 μg/mL, Thermofisher), anti-Iba1 (019-19741 0.5 mg/mL, FUJIFILM Wako) or anti-Ad5 (Polyclonal 1.25 μg/mL, Abcam)] with PBS + Triton 0.1% + bovine serum albumin (BSA) 0.2%. After the washing (PBS triton 0.1%), the samples were incubated with biotinylated anti-rabbit/mouse secondary antibody (VECTOR, R.T.U. VECTASTAIN Kit) for 30 min. The samples were then incubated for another 30 min with the kit’s ABC reagent and revealed with DAB (peroxidase substrate, Vector). After this procedure, counterstaining with hematoxylin (Harris Hematoxylin solution, PanReac AppliChem) and dehydration with alcohols (ethanol 50%, 70%, 96%, 100% and xylol) were performed before the preparations were mounted with DPX medium. Four representative images from each sample were taken, and the positive area in the tissue was quantified by an experienced technician using ImageJ software [41]. 

### 4.6. ICOCAV15 DNA Extraction and Quantification

Whole blood samples were collected during follow-up and stored at −20 °C until processing. DNA was extracted from 200 µL of peripheral blood using the QIAamp DNA Blood Mini Kit (Qiagen, Hilden, Germany). CSF samples were collected on day 21, and months 6, 12 and 18 after the treatment and were stored frozen at −80 °C until processed. A 200-µL portion of each CSF sample was used to extract DNA with PureLink Viral RNA/DNA Mini Kit (Invitrogen, Waltham, MA, USA). The tissues obtained at necropsy (liver, spleen, kidneys, and brain tissues) were processed immediately after tissue extraction. Thirty to 35 mg from each tissue was lysed for 1.5 h at 55 °C, and DNA was extracted using E.Z.N.A.^®^ Tissue DNA kit (Omega Biotech, Norcross, GA, USA). All of the extractions were performed according to the instructions provided by each manufacturer. DNA quantification and purity (A260/280 and A260/230) were analyzed with a Nanodrop 2000 spectrophotometer (Thermo Scientific, Waltham, MA, USA), and DNA samples were stored at −20 °C until further use.

DNA isolated from whole blood, CSF and tissues obtained during necropsy was analyzed by qPCR to detect viral particles of ICOCAV15 in the samples. A standard curve was performed based on serial dilutions of ICOCAV15 from 10^7^ to 10^3^ viral particles (vp)/mL (or 4 × 10^4^ to 4 vp/well), and qPCR was performed using the QuantStudio 3 Real-Time PCR System (Applied Biosystems, Waltham, MA, USA). DNA samples were analyzed in triplicate with a TaqMan system using Premix Ex Taq (Clontech Laboratories Inc, Mountain View, CA, USA), forward primer (0.5 μmol/L) 5′-TGTGGGCCTGTGTGATTCCT-3′, reverse primer (0.5 μmol/L) 5′-CCAGAATCAGCCTCAGTGCTC-3′, and 10 pmol of Taqman probe FAM-CTCGAATCAGTGTCAGGCTCCGCA-TAMRA. The qPCR protocol consisted of a 10-min holding stage at 95 °C and a cycling stage (repeated 40 times) of 15 s at 95 °C followed by 1 min at 60 °C. The results were analyzed using QuantStudio 3 Software (Applied Biosystems, Waltham, MA, USA).

### 4.7. Antiviral Antibodies

The antibodies against canine adenovirus (α-CAV2) were determined from the plasma samples by an enzyme-labeled dot assay (Canine VacciCheck, Antibody Test Kit, Eurovet Veterinaria), following the manufacturer’s instructions. The images were digitized, and the spot densities were quantified using ImageJ software [41]. Arbitrary units were calculated as follows: sample spot density—control positive spot density—background spot density.

## Data Availability

Not applicable.
